# A rapid and flexible microneutralization assay for serological assessment of influenza viruses

**DOI:** 10.1111/irv.13141

**Published:** 2023-04-26

**Authors:** Kalee E. Rumfelt, William J. Fitzsimmons, Rachel Truscon, Arnold S. Monto, Emily T. Martin, Adam S. Lauring

**Affiliations:** ^1^ Department of Epidemiology University of Michigan Ann Arbor Michigan USA; ^2^ Division of Infectious Diseases, Department of Internal Medicine University of Michigan Ann Arbor Michigan USA; ^3^ Department of Microbiology and Immunology University of Michigan Ann Arbor Michigan USA

**Keywords:** hemagglutination, influenza virus, neutralization, serology

## Abstract

**Background:**

Serological responses from influenza vaccination or infection are typically measured by hemagglutinin inhibition (HAI) or microneutralization (MN). Both methods are limited in feasibility, standardization, and generalizability to recent strains. We developed a luciferase MN (LMN) assay that combines the advantages of the conventional MN assay with the ease of the HAI assay.

**Methods:**

Sera were obtained from the HIVE study, a Michigan household cohort. Reverse genetics was used to generate recombinant influenza viruses expressing the hemagglutinin and neuraminidase of test strains, all other viral proteins from an A/WSN/1933 backbone, and a NanoLuc reporter. Serum neutralization of luciferase‐expressing targets was quantified as a reduction in light emission from infected cells. Neutralization titers were measured for cell‐ and egg‐adapted versions of A/Hong Kong/4801/2014 and A/Singapore/INFIMH‐16‐0019/2016 and compared to HAI titers against egg‐grown antigens.

**Results:**

Three hundred thirty‐three sera were collected from 259 participants between May 2016 and July 2018. Sampled participants were 7–68 years of age, and >80% were vaccinated against influenza. HAI and LMN titers were correlated for A/Hong Kong/4801/2014 (*ρ* = 0.52, *p* ≤ 0.01) and A/Singapore/INFIMH‐16‐0019/2016 (*ρ* = 0.79, *p* ≤ 0.01). LMN titers were lower for cell strains compared to egg strains (A/Hong Kong/4801/2014 mean log_2_ fold change = −2.66, *p* ≤ 0.01 and A/Singapore/INFIMH‐16‐0019/2016 mean log_2_ fold change = −3.15, *p* ≤ 0.01).

**Conclusions:**

The LMN assay was feasible using limited sample volumes and able to differentiate small antigenic differences between egg‐adapted and cell‐derived strains. The correspondence of these results with the commonly used HAI confirms the utility of this assay for high‐throughput studies of correlates of protection and vaccine response.

## 1 INTRODUCTION

Influenza causes 9 million to 36 million illnesses and 4000 to 60 000 deaths in the United States (US) each year.^1^ Vaccination is one of the best ways to prevent influenza infection and the large public health burden associated with it.^2^, ^3^ Influenza vaccination has been shown to have variable effectiveness from year to year, a phenomenon that has been attributed to egg‐adaptations occurring during the manufacturing process that hamper the mounting of antibodies to circulating strains.^4^, ^5^, ^6^, ^7^, ^8^, ^9^ For this reason, it is critical to supplement observational studies investigating vaccine effectiveness, with serological analysis of vaccinated and unvaccinated individuals that are able to distinguish between antibodies elicited by egg versus cell‐grown antigens.

Serological responses from influenza vaccination or infection can be investigated through multiple assays.^10^, ^11^, ^12^ The hemagglutinin inhibition (HAI) assay measures the ability of antibodies to inhibit the binding of the virus's hemagglutinin (HA) surface glycoprotein to sialic acids on the surface of red blood cells (RBCs), thereby preventing hemagglutination.^10^ The HAI assay is relatively simple and inexpensive, and it has been considered a gold standard of influenza serology for decades.^10^, ^11^, ^13^ However, HAI assays are vulnerable to nonspecific interfering factors, rely on availability of mammalian or avian fresh red blood cells, have a subjective readout method, and have limited utility for newer H3N2 strains.^10^, ^11^, ^12^, ^13^, ^14^ More recently, microneutralization (MN) assays and focus reduction neutralization tests (FRNT) have become more common, as they are more sensitive than HAI assays and more broadly measure the neutralizing activity of sera.^11^, ^12^, ^14^ The MN assay also performs well across more recent H3N2 strains.^13^ Although MN assays have advantages over the HAI assay, they are more labor intensive and protocols are not well standardized across laboratories.^11^, ^12^, ^14^

Here, we describe a luciferase‐based MN (LMN) assay that combines the advantages of the conventional MN assay with the ease of the HAI assay. We generated recombinant influenza virus targets that express a luciferase reporter, providing a sensitive and quantitative read‐out of cellular infection and the inhibitory activity of sera. We demonstrate this assay's performance using cell culture‐ and egg‐adapted versions of two recent H3N2 strains. We related the LMN assay's detection of neutralization activity against egg‐adapted H3N2 targets to the HAI assay detection of seroreactivity for the same targets and sera. The cell‐adapted targets were used to compare the cell‐adapted and the egg‐adapted LMN neutralization responses. Together, our results demonstrate the utility of this assay for studies of immune correlates of protection.

## 2 METHODS

### 2.1 Participants and sera

Sera were provided from individuals enrolled in the Household Influenza Vaccine Evaluation (HIVE) study. The HIVE study was approved by the Institutional Review Board at the University of Michigan Medical School (HUM00198212), and written informed consent was provided by patients or a proxy/surrogate. Methods pertaining to the HIVE study can be found elsewhere.^15^ Briefly, individuals were followed and contacted weekly to identify acute respiratory infections (ARI). Blood draws were scheduled at enrollment and twice annually after that. Serum was separated via centrifugation. The sera were stored at minus 20°C until further processing. In total, we used 333 serum samples from 259 individuals between May 2016 and July 2018.

### 2.2 HAI assays

All serum samples underwent HAI testing as a standard processing method. Samples were treated with receptor‐destroying enzyme (RDE) following the manufacturer's instructions.^16^ The HAI assays were performed in 96‐well plates by combining a standardized quantity of HA from specific A/H3N2, A/H1N1, and B vaccine strains with serially diluted sera and turkey red blood cells as previously described.^15^ HA targets were obtained through the International Reagent Resource (IRR) and from Sanofi.

### 2.3 Molecular cloning

Viral stocks for A/Hong Kong/4801/2014 (FR‐1453) and A/Singapore/INFIMH‐16‐0019/2016 (FR‐1590) strains were obtained from IRR. Stocks were passaged once at low multiplicity on MDCK‐SIAT1 cells, and RNA was extracted using the QIAmp Viral RNA mini kit (Qiagen). Genomic segments encoding the HA and NA proteins were amplified by reverse transcription polymerase (RT‐PCR) with primers 5′‐TATTCGTCTCAGGGAGCAAAAGCAGGGG‐3′ and 5′‐ATATCGTCTCGTATTAGTAGAAACAAGGGTGTTTT‐3′ for HA and primers 5′‐TATTGGTCTCAGGGAGCAAAAGCAGGAGT‐3′ and 5′‐ATATGGTCTCGTATTAGTAGAAACAAGGAGTTTTTT‐3′ for NA. These primers contain BsmBI and BsaI restriction sites to facilitate cloning into pHW2000 (a gift from Robert Webster, St. Jude Children's Research Hospital). The egg‐adapted sequences of these plasmid clones were verified by Sanger sequencing. The egg‐adapted A/Hong Kong/4801/2014 strain contained four point mutations in the HA open reading frame and one point mutation in the NA open reading frame. The egg‐adapted A/Singapore/INFIMH‐16‐0019/2016 strain contained three point mutations in HA and no point mutations in NA. To create the cell‐adapted strains, gene synthesis was performed by Integrated DNA Technologies (IDT) for A/Hong Kong/4801/2014 (HA and NA) and A/Singapore/INFIMH‐16‐0019/2016 (only HA). The synthesized segments contained BsmBI and BsaI restriction sites to facilitate cloning into pHW2000. The cell‐adapted sequences of these plasmid clones were verified by Sanger Sequencing.

### 2.4 Rescue of recombinant luciferase expressing influenza viruses

For generation of luciferase reporter recombinant viruses by reverse genetics, 5 × 10^4^ HEK 293T and 2.5 × 10^4^ MDCK‐SIAT1 cells were plated in 12‐well plates in growth media (DMEM, Invitrogen #19965‐092, with a final concentration of 10% fetal bovine serum [FBS, Invitrogen #26140‐079], 100 units/mL penicillin [Invitrogen #15140‐122], 100ug/mL streptomycin [Invitrogen #15140‐122], 2‐mM L‐glutamine [Invitrogen #25030‐081]) the day prior to transfection.^17^ These co‐cultures were transfected with 500 ng each of PASTN‐PA‐NanoLuc (a gift from Andrew Mehle, University of Wisconsin), strain specific pHW184‐HA, strain specific pHW186‐NA, pHW181‐PB2, pHW182‐PB1, pHW185‐NP, pHW187‐M, pHW188‐NS, and TransIT‐LT1 (MIRUS #MIR2300). The PB2, PB2, NP, M, and NS segments were on A/WSN/1933 plasmid backbones. After 12 h, the cultures were washed and seeded with viral media (DMEM, Invitrogen #19965‐092, with a final concentration of 0.1875% bovine serum albumin [BSA, Invitrogen #15260‐037], 25‐mM HEPES [Invitrogen #15630‐080], 100 units/mL penicillin [Invitrogen #15140‐122], 100 μg/mL streptomycin [Invitrogen #15140‐122], 0.1 μg/mL TPCK‐treated trypsin [Worthington Biochemical #LS003740]). Once full cytopathic effect was observed, supernatants were centrifuged at 1400×*g* for 4 min and stored in a final concentration of 0.5% glycerol at −80°C. These passage 0 stocks were titered and used to infect 1 × 10^6^ MDCK‐SIAT1 at an MOI of 0.01. These passage 1 supernatants were harvested as above and stored at −80°C in single use aliquots. Stocks were titered as median tissue culture infectious dose (TCID_50_) in 96‐well plates, by scoring positive wells as luciferase expression that exceeded two times the cellular background. This titering method was consistent with conventional TCID_50_ measurements that scored cytopathic effect.

### 2.5 LMN assay

Human (positive) control serum was obtained from a vaccinated donor and stored at −20°C. Lyophilized sheep (negative) control serum was obtained from IRR, reconstituted using sterile water, and stored at −20°C. Before use, all serum aliquots were thawed at room temperature and resuspended by vortexing. Human control, sheep control, and patient sera were heat inactivated (HI) at 56°C for 30 min but not treated with RDE prior to use.

**FIGURE 1:**
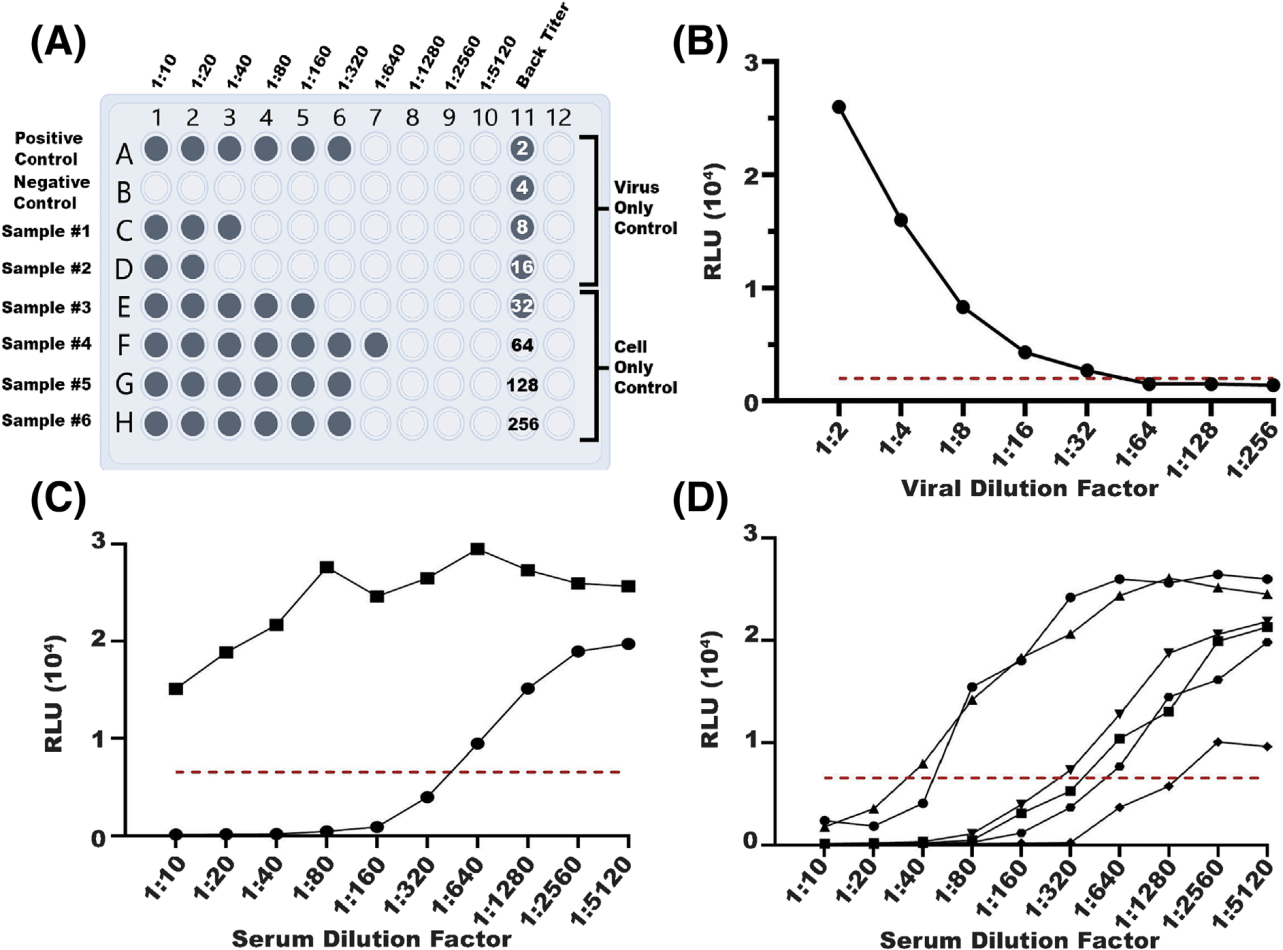
Design of the luciferase microneutralization assay (LMN). (A) Plate layout: Sera wells (rows A‐H, columns 1–10) are considered positive for 75% neutralization of virus if the relative light unit (RLU) output is above the virus control RLU average minus the cell control RLU average divided by 4. Numbers in back titer wells indicate fold dilution of the input virus (100 infectious units). A back titer well is considered positive if the RLU output was greater than twice the cell only control RLU average. A plate was considered “passing” if the first negative well fell between the fifth and eighth dilution (1:32 to 1:256). Positive sera and back titer wells are colored. (B) Example of back titer with RLU (*y*‐axis) for each virus dilution (*x*‐axis). A visible leveling off in back titer between the fifth and eighth dilution was required for a plate to pass quality control. The red line denotes the cutoff of the cell only control average multiplied by two. (C) Example of positive (human) neutralization control (circles) and negative (sheep) neutralization control (squares). The neutralization cutoff, based on 75% reduction in RLU, is denoted by a red line. (D) Example of titrations for six sera, each denoted by a different shape. The 75% cutoff for neutralization is denoted by a red line.

Control and patient sera were added to column 1 at a volume of 10 μL and serially diluted across the plate in 100‐μL total volume (Figure 1A). For each target virus, the P1 stock was diluted to a concentration of 2 × 10^3^ TCID50/mL and 50 μL (100 infectious units) were added to all the serum dilution wells. A back titer series was created by adding to 50 μL (100 infectious units) to the first well in a column and then performing twofold serial dilutions down to the last well. After a 1‐h incubation at 37°C, 100‐μL MDCK‐SIAT1 cells at a concentration of 1.5 × 10^5^ cells/mL were added to each well. The plate was then incubated for 18 h at 37°C.

After incubation, the supernatants were aspirated from the wells and 50 μL of a 1:8000 concentration of ViviRen (Promega, #E6491) in viral media were added to each well. The plate was incubated at room temperature for 3 min, and luminescence was measured in a Synergy HTX multi‐mode luminometer using a gain of 160. All the steps involving ViviRen were performed in a darkened room.

### 2.6 Statistical analysis

Spearman's rank correlation coefficients (SRCC) were calculated to determine correlations between LMN and HAI titers separately for the A/Hong Kong/4801/2014 and the A/Singapore/INFIMH‐16‐0019/2016 strains. Paired *t* tests were used to compare LMN neutralization titer against the egg‐ versus cell‐adapted strains of A/Hong Kong/4801/2014 and A/Singapore/INFIMH‐16‐0019/2016. All statistical analyses were performed using SAS software version 9.4 (SAS Institute, Cary, NC).

## 3 RESULTS

The LMN assay is modeled after a traditional MN assay, which measures neutralization of virus in the presence of serum with potentially protective antibodies. In the LMN, influenza virus targets express the NanoLuc reporter from segment 3 (encoding PA). We used standard influenza virus reverse genetics to generate recombinant 6 + 2 viruses with segments expressing the hemagglutinin (HA) and neuraminidase (NA) of test strains and all other proteins, including the reporter, from A/WSN33 (H1N1). Expression of the NanoLuc reporter was sufficient to detect a single infectious event at 18 h post‐infection, as titers of viral stocks measured by luminescence over background produced similar titers measured by cytopathic effect (NanoLuc TCID50 vs. TCID50, Table S1). All viruses replicated to high titer in MDCK cells.

As in the traditional MN assay, the LMN assay is performed in 96‐well plates with 100 infectious units of target virus in each well and serial dilutions of test sera (Figure 1A).^11^ The plate has dedicated wells for “virus only controls (VC)” to capture maximum luminescent output, “cell only controls (CC)” to measure background, and twofold serial dilutions of the target virus as a “back titer” to ensure that 100 infectious units were used. For a plate to pass quality control, the luminescent output (RLU) in the back titer wells should be less than two times the CC average in at least one well between wells 5 and 8 (viral dilutions of 1:32–1:256).

The 75% neutralization titer (NT_75_) is calculated as the serum dilution at which luminescence is reduced 75% relative to the VC (maximum luminescence in the absence of serum) and CC (background cellular luminescence) wells (Figure 1A). In preliminary testing, we found that the 75% cutoff was more discriminatory than a 50% cutoff given cellular background and occasional flat, as opposed to sigmoidal, curves with non‐neutralizing control sera.

We evaluated the performance of the LMN assay using 333 serum specimens from 259 individuals collected before and after the 2016/2017 and/or the 2017/2018 Northern hemisphere influenza seasons. Thirty‐seven individuals had paired preseason and postseason specimens for the same season, and some contributed three or four total specimens (Table 1). These sera were chosen because they exhibited discordant HAI assay results for A/Hong Kong/4801/2014 and A/Singapore/INFIMH‐16‐0019/2016, two related but antigenically distinct, vaccine strains. The sampled individuals ranged from 7 to 68 years of age with many being between 41 and 64 years of age (49%). The sera came from a heavily vaccinated cohort with 81.4% and 86.5% of individuals having received the recommended annual influenza vaccine prior to the 2016–2017 and 2017–2018 influenza seasons, respectively.

**TABLE 1:** Demographic characteristics, vaccination history, and infection history of sampled individuals.

	May 2016–August 2016	November 2016–January 2017	May 2017–August 2017	September 2017–December 2017	May 2018–July 2018
	(*n* = 44)	(*n* = 43)	(*n* = 43)	(*n* = 89)	(*n* = 40)
H3N2 vaccine/circulating strain	A/Switzerland/9715293/2013	A/Hong Kong/4801/2014	A/Hong Kong/4801/2014	A/Hong Kong/4801/2014	A/Hong Kong/4801/2014
**Age** ^**a**^					
7–12 years	1 (2.3)	‐	‐	10 (11.2)	‐
13–18 years	5 (11.3)	5 (11.6)	5 (11.6)	15 (16.9)	4 (10.0)
19–25 years	1 (2.3)	2 (4.7)	2 (4.7)	3 (3.4)	1 (2.5)
26–40 years	16 (36.4)	12 (27.9)	12 (27.9)	26 (29.2)	11 (27.5)
41–64 years	21 (47.7)	24 (55.8)	24 (55.8)	34 (38.2)	24 (60.0)
65+ years	‐	‐	‐	1 (1.1)	‐
**Received seasonal vaccine** ^**a**^		35 (81.4)		77 (86.5)	

^a^ Age, vaccination history, and infection history are presented as *n* (%). Vaccination history was assessed for vaccination in the previous season. One person could have given multiple samples within the same season and across seasons (subject *n* = 259, sample *n* = 333).

**FIGURE 2:**
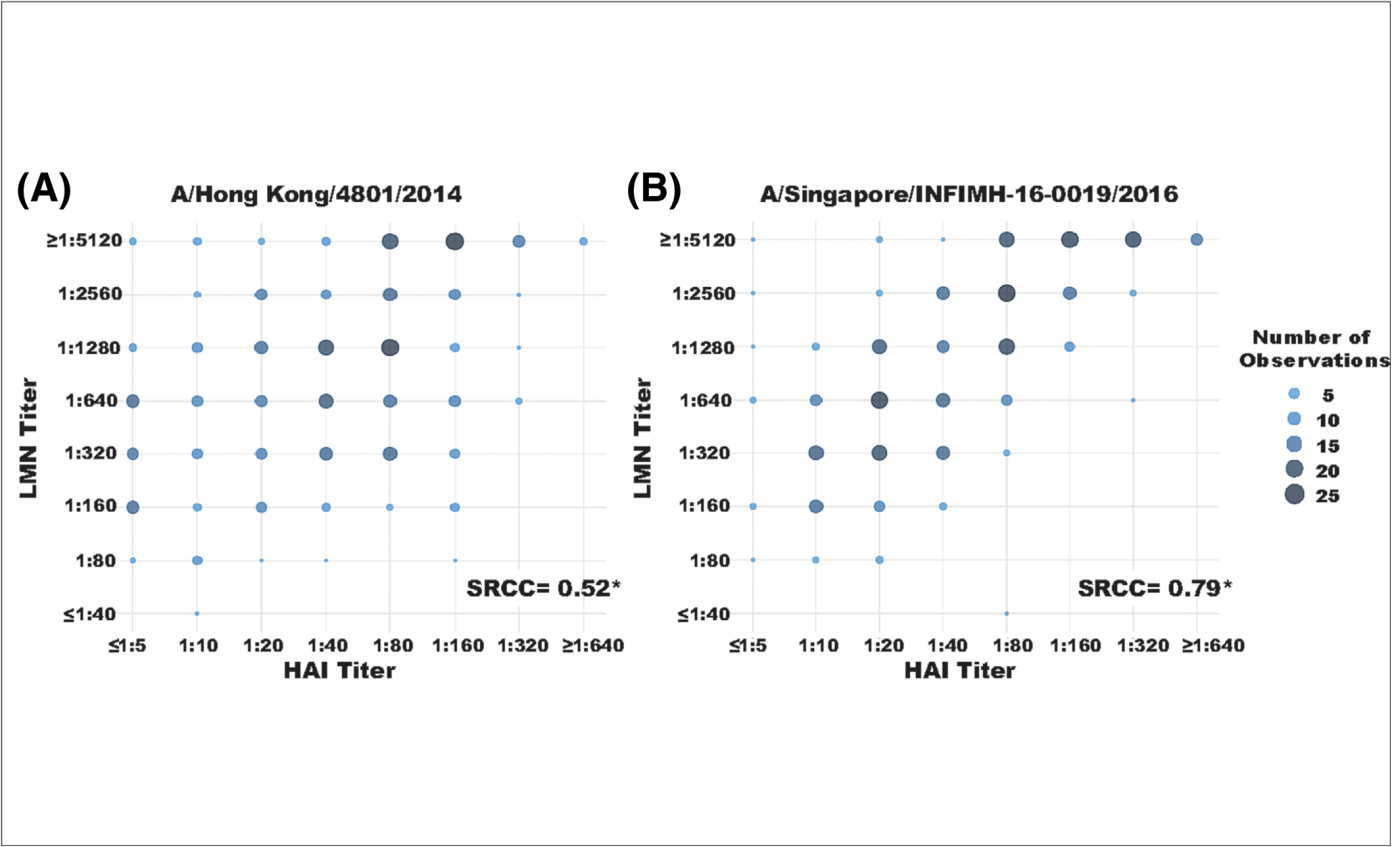
Correlation between HAI and LMN NT_75_ for (A) A/Hong Kong/4801/2014 and (B) A/Singapore/INFIMH‐16‐0019/2016; *n* = 333 *Spearman's Rank Correlation Coefficients with a *p* value ≤0.01.

We compared LMN NT_75_ to HAI titer for each of these sera. Because these assays differ in their sensitivity, specificity, and dynamic range, both were ordinalized using a common approach. The NT_75_ for a negative control serum (e.g., sheep serum) was set to 0 and each twofold serial dilution of the test sera was therefore 1, 2, 3, and so forth. For example, a LMN result with a negative control NT_75_ of 1:40 and a test serum NT_75_ of 1:160 would be expressed as 2. After ordinalization, we found that the HAI titer and the LMN NT_75_ were significantly correlated, but the magnitude differed by strain. Titers were moderately correlated for A/Hong Kong/4801/2014 (SRCC 0.52, *p* ≤ 0.01; Figure 2A) and strongly correlated for A/Singapore/INFIMH‐16‐0019/2016 (SRCC 0.79, *p* ≤ 0.01; Figure 2B).

**FIGURE 3:**
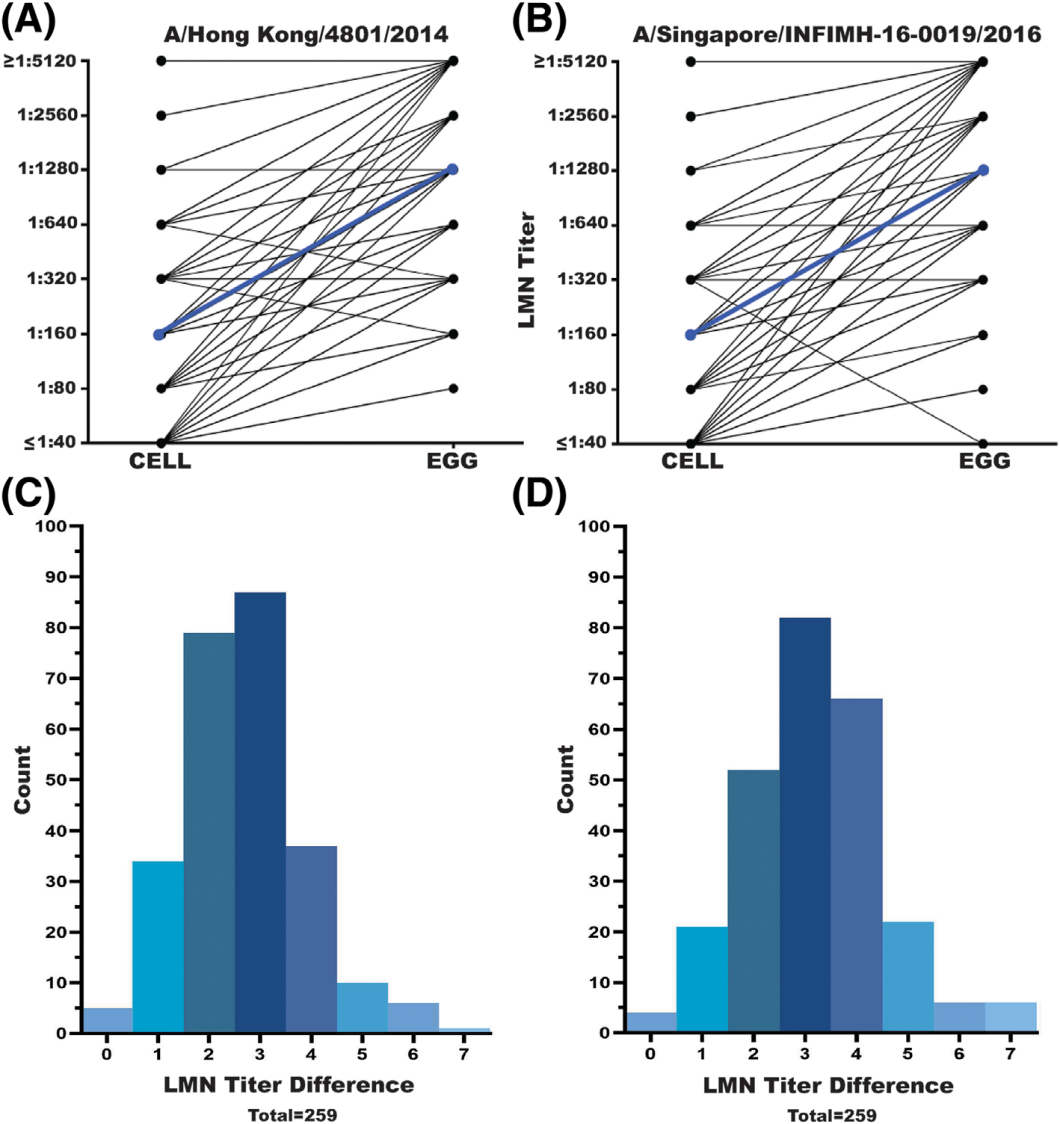
Difference in paired profile of LMN NT_75_ across egg‐ and cell‐ adapted influenza viruses. (A,B) Mean log_2_ fold change in LMN NT_75_ from cell‐ to egg‐adapted viral targets is denoted by a blue line. Both the Hong Kong and Singapore strains showed a statistically significant different LMN NT_75_ for cell‐ and egg‐adapted virus strains (Hong Kong t‐value = −34.36, dF = 258, *p* value ≤0.01; Singapore *t* value = −36.26, dF = 258, *p* value ≤0.01). (C) and (D) Mean log_2_ fold difference in LMN NT_75_ across cell‐ and egg‐adapted virus strains (Hong Kong mean log_2_ fold change = −2.66 [−2.82, −2.51]; Singapore mean log_2_ fold change = −3.15 [−3.33, −2.98]). All individuals had higher LMN NT_75_ for the egg‐adapted strains except two individuals with a onefold difference for the A/Hong Kong/4801/2014 and one individual with a threefold difference for A/Singapore/INFIMH‐16‐0019/2016.

We measured LMN NT_75_ against cell culture‐ and egg‐adapted A/Hong Kong/4801/2014 and A/Singapore/INFIMH‐16‐0019/2016 influenza viruses to determine if the LMN assay could detect antigenic differences due to the corresponding mutations in HA and NA. We created egg‐adapted versions of these strains by using HA and NA segments that corresponded to sequences of egg passaged viruses (see Methods). Because we rescued and propagated these egg‐adapted strains in MDCK cells, we sequenced all stocks to ensure that the egg‐ and cell culture‐associated mutations were maintained. The LMN detected differences in ordinalized LMN NT_75_ for cell culture‐ and egg‐adapted strains of A/Hong Kong/4801/2014 (mean log_2_ fold change = −2.66; Figure 3A,C) and A/Singapore/INFIMH‐16‐0019/2016 (mean log_2_ fold change = −3.15; Figure 3B,D). All sera had higher NT_75_ for the egg‐adapted strains except two sera against A/Hong Kong/4801/2014 and one serum against A/Singapore/INFIMH‐16‐0019/2016 (Figure 3).

## 4 DISCUSSION

In this study, we present a new luciferase‐based MN assay that performs similarly to the gold‐standard HAI assay. We observed significant correlations between the LMN NT_75_ and HAI titer for two recent H3N2 strains, A/Hong Kong/4801/2014 and A/Singapore/INFIMH‐16‐0019/2016. Additionally, we were able to show that the LMN assay can detect differences in LMN NT_75_ for genetically similar egg‐adapted and cell‐adapted influenza viruses. Our results demonstrate that the LMN assay is a promising alternative to the HAI assay and is applicable to a broader range of research questions in different populations, including the study of subtle antigenic differences between strains that may have a major impact on seroprotection.

HAI titers are widely used as a serologic correlate of protection, although some have expressed concerns regarding their utility.^12^, ^13^, ^18^, ^19^, ^20^ The LMN holds key advantages over the HAI assay. Specifically, the HAI assay has a subjective readout method, which can limit reproducibility, and it can produce variable results using recent H3N2 influenza strains.^13^ The LMN assay addresses reproducibility issues by using the back titer, a built‐in standardization component that provides a secondary check to ensure that the proper concentration of virus was added to each well on the plate.^11^ If the back titer fails, the assay results are invalid and the assay must be repeated. This prevents extrapolating information from plates that were subject to experimental error. Additionally, the readout from the luminometer and calculation of the NT_75_ is performed objectively using pre‐specified RLU cutoffs, eliminating subjective assessments of positive vs. negative wells. Another promising attribute of the LMN is that it performs well for many recent H3N2 strains (e.g. A/Hong Kong/4801/2014), which do not hemagglutinate adequately in standard HAI assays.^13^

Although the LMN assay is similar to the traditional MN assay, it is easier to perform and quicker than the original. Generating target viruses for the LMN is relatively straightforward using standard influenza virus reverse genetics with cloned HA and NA genes, and targets can be produced in cells or eggs.^21^ This allows for faster, more efficient virus propagation and flexibility in choosing influenza target strains. The LMN assay can be performed in as few as 20 h from start to finish expediting research efforts, where traditional MN assays can take up to 4 days to complete. The luminometer takes only a few minutes to produce a readout, much shorter than staining procedures during MN.

The LMN assay and the HAI assay measure slightly different antibody activities. Whereas the HAI assay quantifies inhibition of hemagglutination, a proxy of virus‐cell binding, the LMN assay measures inhibition of cell entry, a proxy of neutralization.^10^, ^11^ The LMN assay is more sensitive and has a broader dynamic range compared to the HAI assay (Figure S1). For this reason, we standardized LMN NT_75_ to the range of HAI titers by expressing the LMN NT_75_ as ordinal values and setting our “first” titer to the value one titer greater (1:40) than the negative control output (1:20). This allowed for better comparisons across assays. After standardization, we found that LMN NT_75_ and HAI titer are well correlated, suggesting that the LMN assay is a viable alternative to the HAI assay. We did find that the correlation was lower for A/Hong Kong/4801/2014 compared to A/Singapore/INFIMH‐16‐0019/2016. These differing correlations can potentially be explained by altered sialic acid binding and hemagglutination properties of the A/Hong Kong/4801/2014 HA, highlighting the advantage of using a cell‐based LMN assay.^13^

The LMN assay has several limitations. While molecular cloning and reverse genetics have been widely used to generate virus, these techniques may not be accessible to some surveillance laboratories. Furthermore, these lab‐generated viral strains do have key differences compared to circulating viruses. For example, the viruses we created are on a WSN33 backbone with a luciferase reporter linked to the PA segment and contain cloned HA and NA segments. With these key differences, there is potential for inconsistencies in antibody binding to these strains compared to the same strains found in nature. However, all HA and NA segments were verified via Sanger sequencing and these methods have been shown to be an acceptable alternative to using primary isolates.^13^, ^21^, ^22^ Additionally, a luminometer is needed to perform this assay, which is an additional expense over the HAI assay. The validation study itself also was subject to limitations. First, our egg‐adapted influenza stocks were propagated on MDCK‐SIAT1 cells and not chicken eggs. The cellular glycans are different from those in eggs. However, we sequenced our final stocks of virus to ensure that our “egg‐adapted” stocks still contained the previously documented egg‐adapted genetic changes.^7^, ^23^, ^24^ The LMN assay also was able to detect twofold to threefold differences in NT_75_ for egg‐ and cell‐adapted viruses, which suggests further utility for a wide‐range of differing influenza targets. Lastly, we are unable to draw conclusions regarding outside factors that may impact protection toward influenza. This study was performed for assay development and validation, with the samples being selected based on discordant HAI titer results to differing H3N2 influenza viruses (data not shown). Because of potential bias, we cannot draw conclusions from these data related to the impact of vaccination or infection history on HAI titer or LMN NT_75_. However, our results provide confidence that this method has the resolution needed to further delve into the impacts of vaccination and infection history on protection.

In summary, the LMN assay was able to produce similar results to the gold‐standard HAI assay, as well as detect differences in neutralization response for egg‐ and cell‐adapted influenza strains. Together, these results demonstrate the utility of this assay for studies of immune correlates of protection and highlight key antigenic differences between egg‐adapted vaccine strains and their circulating counterparts.

## AUTHOR CONTRIBUTIONS

Kalee E. Rumfelt, William J. Fitzsimmons, and Rachel Truscon performed all experiments. Emily T. Martin and Arnold S. Monto provided resources. Emily T. Martin, Arnold S. Monto, and Adam S. Lauring obtained funding and provided supervision. Kalee E. Rumfelt and Adam S. Lauring drafted the manuscript. Kalee E. Rumfelt, Arnold S. Monto, Emily T. Martin, and Adam S. Lauring revised and finalized the manuscript.

## CONFLICT OF INTEREST STATEMENT

Emily Martin has received research funding from Merck, unrelated to the submitted work. Arnold Monto and Adam Lauring have received consulting fees from Roche, unrelated to the submitted work.

### PEER REVIEW

The peer review history for this article is available at https://www.webofscience.com/api/gateway/wos/peer-review/10.1111/irv.13141.

## Supporting information


**Figure S1.** Comparison of HAI titer and LMN NT_75_ before and after LMN NT_75_ adjustment. (A) and (B) HAI titer is denoted by the orange line and unadjusted LMN NT_75_ is denoted by the blue line. (C) and (D) HAI titer is denoted by the orange line and adjusted LMN NT_75_ is denoted by the blue line. Corresponding ordinal HAI titer and true LMN NT_75_ values are listed in the middle of the unadjusted A/Hong Kong and A/Singapore comparisons; corresponding ordinal HAI titer and adjusted LMN NT_75_ values are listed in the middle of the adjusted A/Hong Kong and A/Singapore comparisons

**Table S1.** TCID50 and nanoluc TCID50 titers for nanoluc test strains.

## Data Availability

All data are available upon reasonable request by contacting the corresponding author.
