# Supplementary figures and images for: A rapid and flexible microneutralization assay for serological assessment of influenza viruses
Source: Influenza Other Respir Viruses. 2023 Apr 26;17(4):e13141. doi: 10.1111/irv.13141 (PMC10174083; doi:10.1111/irv.13141)

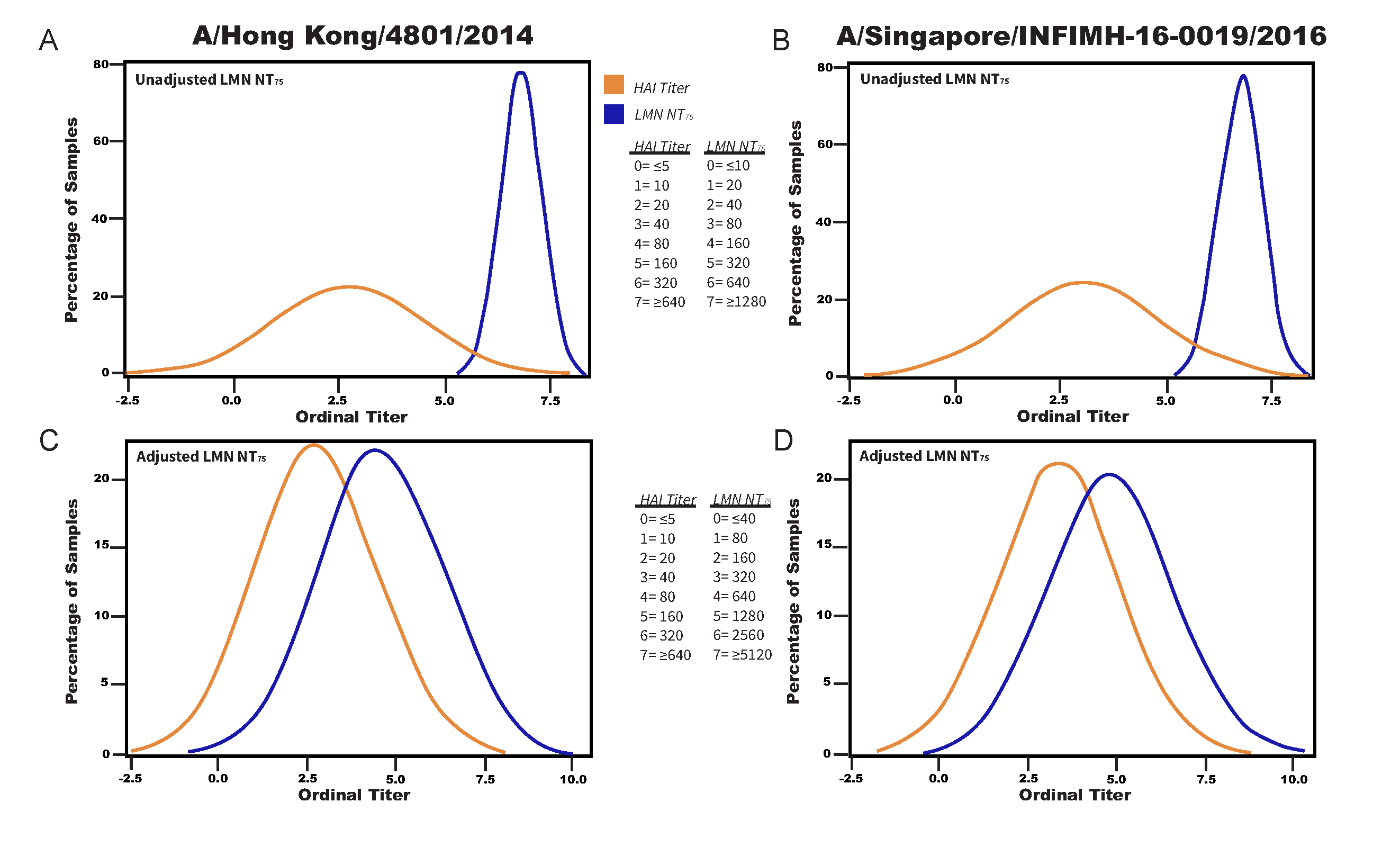

Supplement: Supplementary file 1 — Figure S1. Comparison of HAI titer and LMN NT75 before and after LMN NT75 adjustment. (A) and (B) HAI titer is denoted by the orange line and unadjusted LMN NT75 is denoted by the blue line. (C) and (D) HAI titer is denoted by the orange line and adjusted LMN NT75 is denoted by the blue line. Corresponding ordinal HAI titer and true LMN NT75 values are listed in the middle of the unadjusted A/Hong Kong and A/Singapore comparisons; corresponding ordinal HAI titer and adjusted LMN NT75 values are listed in the middle of the adjusted A/Hong Kong and A/Singapore comparisons [file IRV-17-e13141-s001.tif]
