# Supplementary material for: A rapid and flexible microneutralization assay for serological assessment of influenza viruses
Source: Influenza Other Respir Viruses. 2023 Apr 26;17(4):e13141. doi: 10.1111/irv.13141 (PMC10174083; doi:10.1111/irv.13141)
Supplement: Supplementary file 2 — Table S1. TCID50 and nanoluc TCID50 titers for nanoluc test strains. [file IRV-17-e13141-s002.docx]

**Supplemental Table 1.** TCID50 and nanoluc TCID50 titers for nanoluc test strains.

| **Luciferase Virus** | **Conventional CPE Titer** | **ViviRen RLU Titer** |
| --- | --- | --- |
| A/WSN/1933 | 2.85x10^5^ | 5.62x10^5^ |
| A/Wisconsin/67/2005 | 2.74x10^6^ | 3.98x10^6^ |
| A/Hong Kong/4801/2014 | 3.51x10^6^ | 1.78x10^7^ |
| A/Singapore/INFIMH-16-0019/2016 | 4.14x10^6^ | 1.78x10^7^ |
| A/Ann Arbor/14/2018 | 2.71x10^6^ | 3.16x10^6^ |
